# Epidemiology of neuromyelitis optica spectrum disorder in Denmark (1998–2008, 2007–2014)

**DOI:** 10.1002/brb3.1338

**Published:** 2019-06-11

**Authors:** Nasrin Asgari, Soeren T. Lillevang, Hanne P. B. Skejoe, Kirsten O. Kyvik

**Affiliations:** ^1^ Department of Regional Health Research Odense Denmark; ^2^ Department of Molecular Medicine University of Southern Denmark Odense Denmark; ^3^ Department of Clinical Immunology Odense University Hospital Odense Denmark; ^4^ Department of Radiology Aleris‐Hamlet Hospital Copenhagen Denmark; ^5^ OPEN (Odense Patient data Explorative Network) Odense University Hospital Odense Denmark; ^6^ Department of Clinical Research University of Southern Denmark Odense Denmark

**Keywords:** epidemiology, neuromyelitis optica spectrum disease

## Abstract

Epidemiological studies of the uncommon disorder neuromyelitis optica spectrum disorder **(**NMOSD) may be difficult to interpret because of the evolving nature of diagnostic criteria, differences in the definition and accuracy of NMOSD diagnosis, the completeness of case ascertainment, and variability in assays for the disease‐specific biomarker aquaporin‐4 (AQP4)‐IgG. A sub‐group of patients with the clinical syndrome NMOSD lack detectable AQP4‐IgG and in these cases an accurate diagnosis requires precise diagnostic algorithms and longitudinal follow‐up. Consecutive sets of criteria for NMO/NMOSD have been introduced during the two last decades. Such criteria need validation in different populations. Detection of other autoantibodies, such as IgG specific for myelin oligodendrocyte glycoprotein or for glial fibrillary acidic protein in a sub‐group of AQP4‐IgG–negative NMOSD patients, has improved over the past decade and may lead to overlap of the clinical syndromes/phenotypes. This review begins by summarizing current knowledge on the widening clinical spectrum of NMOSD. Subsequently, we describe two epidemiological studies from Denmark carried out in two different decades (1998–2008 and 2007–2014) and comment on the differences in study design, patient ascertainment, and interpretation of results. These factors may explain some of the observed differences, reflecting the complexity and providing a clear example of this development.

## 1 INTRODUCTION

Neuromyelitis optica spectrum disorder (NMOSD) is a relapsing inflammatory disease of the central nervous system (CNS) (Weinshenker & Wingerchuk, 2017) and probably the most common of the non‐multiple sclerosis (MS) inflammatory demyelinating diseases (IDDs) of the CNS (Flanagan & Weinshenker, 2014; Jacob et al., 2007). NMOSD is believed to be an autoimmune astrocytopathy, where the damage to astrocytes exceeds the damage to myelin and neurons, in contrast to MS as a mainly myelin‐directed disorder (Kawachi & Lassmann, 2017).

During the past two decades, the definition and diagnostic criteria for NMO/SD have evolved from Devic's clinical description from 1894 into a more heterogeneous clinical presentation (Wingerchuk, Lennon, Lucchinetti, Pittock, & Weinshenker, 2007). Detection of a highly disease‐specific serum autoantibody against the astrocyte water channel aquaporin‐4 (AQP4), and its use as a diagnostic tool, indicates a broader clinical phenotype of this disorder (so‐called NMOSD) leading to recognition of NMOSD as a distinct entity (Wingerchuk et al., 2015). Since NMOSD is a severe CNS IDD with a less favorable prognosis than MS and with a different treatment approach (Trebst et al., 2014), early diagnosis based on robust criteria is critical (Wingerchuk et al., 2015). Three sets of criteria for diagnosis have been proposed (Wingerchuk et al., 2015, 1999; Wingerchuk, Lennon, Pittock, Lucchinetti, & Weinshenker, 2006). Several different immunoassays with various immunological techniques have been developed for the detection of AQP4‐IgG (Waters et al., 2016). Their sensitivities vary considerably, whereas specificities are uniformly high (Jarius et al., 2014; Waters et al., 2014).

Knowledge of NMOSD epidemiology is critical for appropriate allocation of healthcare resources (Weinshenker & Wingerchuk, 2017). Patients with NMOSD have been reported from different regions of the world and from different ethnicities (Pandit et al., 2015). The disease appears to occur more often in populations of African, East Asian, and Latin American descent than in other populations (Mori, Kuwabara, & Paul, 2018; Pandit et al., 2015). However, the diagnostic criteria have not been uniform and different AQP4‐IgG assays have been used, which may explain some of the differences across studies. In addition, most studies have been carried out in small populations based on cases from tertiary hospitals and therefore have an inherent risk of bias (Pandit et al., 2015). We discuss current understanding of the clinical aspects of NMOSD and two epidemiological studies carried out in two different decades, providing a clear example of this complexity.

### 1.1 Diagnostic criteria of NMO/SD

The NMOSD diagnostic criteria have been revised several times during the last two decades, mainly due to improved understanding of AQP4 autoimmunity. Wingerchuk, Hogancamp, O'Brien, and Weinshenker (1999) described diagnostic criteria based on the natural history of NMOSD including demographic and clinical information as well as MRI features (Wingerchuk et al., 1999). However, the criteria from 1999 were not particularly operational because one of the three absolute requirements for NMO diagnosis was the absence of extra‐opticospinal symptoms or CNS symptoms. Furthermore, one requirement was a normal brain MRI or a non‐MS‐like MRI at disease onset (Wingerchuk et al., 1999). As a consequence patients who had evidence of clinical disease involving other regions of the CNS, or who had MS‐like lesions or brainstem lesions, were excluded. These limitations led to a revision of the NMOSD diagnostic criteria based on radiological as well as clinical features and AQP4‐IgG tests (Wingerchuk et al., 2006). As a result, 15% of NMOSD patients had experienced neurological symptoms referable to disease elsewhere in the CNS, furthermore up to 60% had radiological dissemination in space and time, and demonstrated more complex clinical manifestations. These criteria represented a significant improvement compared to previous criteria as they removed the restriction of non‐CNS involvement in addition to the involvement of optic nerves and spinal cord.

Lately, new diagnostic criteria have appeared (International Panel for NMOSD Diagnosis [IPND], 2015) which introduce the unifying term NMOSD (further divided into NMOSD with or without AQP4‐IgG). The IPND criteria supposedly enable diagnosis of NMOSD with both high specificity and sensitivity. This is a matter of key importance as treatment options suitable for MS can be deleterious for patients with NMOSD (Borisow, Mori, Kuwabara, Scheel, & Paul, 2018). Early diagnosis and better distinction of NMOSD from MS are thus expected (Wingerchuk et al., 2015). The diagnostic strategy is based on serological determination of AQP4‐IgG. Based on the core clinical characteristics, a diagnosis may be made after a single characteristic clinical episode in patients, who are positive for AQP4‐IgG (Wingerchuk et al., 2015). A proportion of NMOSD patients are seronegative and must be diagnosed on a purely clinical basis with at least two different clinical episodes documented with MRI, characterized by at least two lesions in separate structures (radiological dissemination) corresponding to the clinical episode. Thus, more stringent clinical criteria and additional neuroimaging findings are required for the diagnosis of NMOSD without AQP4‐IgG (Flanagan et al., 2015; Kim et al., 2015).

Very recently, a large multicenter MRI study has reported a high prevalence of atypical CNS MRI findings in up to 50% of NMOSD patients, such as short transverse myelitis (TM) (36.1%). In total, 37.1% of the patients met the 2010 McDonald criteria for MS (Cacciaguerra et al., 2019). This suggests that, even based on current MRI criteria, there remains a relatively high percentage of NMOSD patients with diagnostic uncertainty, independent on the presence of AQP4‐IgG (Cacciaguerra et al., 2019). If confirmed, the recommended MRI criteria based on the typical MRI findings in NMOSD patients may have to be revised.

Furthermore, recent studies have successfully established the characteristics of optical coherence tomography following AQP4‐IgG‐mediated optic neuritis (ON) which have been shown to be useful in differentiating AQP4‐IgG‐mediated ON from MS (Bennett et al., 2015; Oertel, Zimmermann, Paul, & Brandt, 2018; Pache et al., 2016).

It may be concluded that the progress in clinical and basic research has dramatically expanded the clinical and neuroimaging spectrum of NMOSD. Even so, the most recently proposed diagnostic criteria require validation through large (and preferably multicenter) investigations.

### 1.2 AQP4‐IgG serostatus

AQP4‐IgG–associated NMOSD is defined as an antibody‐mediated disorder with female predominance and a relapsing phenotype (Gold, Willing, Leypoldt, Paul, & Friese, 2019). The high specificity for AQP4‐IgG gives a strong basis for the NMOSD diagnosis. However, quantitative AQP4‐IgG levels do not predict response to therapy (Mealy et al., 2018) or overall long‐term outcome of NMOSD (Kessler et al., 2017). AQP4‐IgG is not detected in 10%–40% of patients diagnosed with NMOSD, (Weinshenker & Wingerchuk, 2017) based on the availability of assays with high specificity and variable sensitivity (Melamed et al., 2015). This limitation constitutes a specific diagnostic challenge. Multiple factors may influence the assay results, such as sex, age, ethnicity, disease activity, immunotherapy, and variations in assay techniques (Melamed et al., 2015). In practical terms, AQP4‐IgG–negative NMOSD patients may be difficult to diagnose despite clinical and imaging evidence (Juryńczyk et al., 2016). Several techniques are currently available to test for serum AQP4‐IgG and can be categorized according to whether they are tissue, cell, or protein‐based (Waters et al., 2014). Seronegative NMOSD may occur for the following reasons: (a) true negativity (i.e., where pathogenic factors other than AQP4‐IgG are involved as mentioned above. IgG specific for myelin oligodendrocyte glycoprotein [MOG‐IgG] was reported in 10%–20% of NMOSD‐suspected but AQP4‐IgG–negative patients [Jarius et al., 2016a, 2016b; Kitley et al., 2014; Mader et al., 2011; Pache et al., 2016; Sato et al., 2014; Soelberg et al., 2017]); (b) differences in assay performance; and (c) testing of a patient on immunosuppressive therapy (which leads to lower or absent antibody levels [Jacob et al., 2007; Waters et al., 2014]). AQP4‐IgG levels have been shown to be higher during relapse as opposed to during remission, and the retesting of initially seronegative patients during an acute attack or during a treatment‐free interval may show positivity (Jarius et al., 2008, 2010). Even so, the absence of AQP4‐IgG leaves the clinician with the possibility of an alternative diagnosis, such as an optic‐spinal form of MS or another IDD of the CNS (Juryńczyk et al., 2016; Weinshenker & Wingerchuk, 2017).

In a recent retrospective case series, 27 neurology consultants with expertise in IDDs of the CNS scored the clinical and radiological data from 12 patients with AQP4‐IgG–negative NMOSD or MS‐like syndromes from the Oxford NMO service (Juryńczyk et al., 2016). The experts frequently disagreed on the diagnosis of AQP4‐IgG–negative NMOSD versus MS in patients (Juryńczyk et al., 2016), indicating that the borders between AQP4‐IgG–negative NMOSD and other IDDs of the CNS are poorly demarcated and that accurate diagnosis may require longitudinal follow‐up.

### 1.3 1998–2008

A population‐based study was performed with an 11‐year observation period (1 January 1998 to 31 December 2008) (Asgari et al., 2011). The study originated from Department of Regional Health Research, University of Southern Denmark. The data originated from multiple sources, including the Danish National Patient Registry (DNPR) (*n* = 2,170), four neurology and three ophthalmology departments (*n* = 1,399), and from a database registering patients with MS treated with natalizumab (*n* = 66) (Asgari et al., 2011). The IDD diagnoses were confirmed by the data list extracted from the DNPR and cross‐checked with the Danish Central Personal Registry (Asgari et al., 2011). In addition, the research service of the Danish Health Board provided updated information on the civil status (dead/alive/emigrated) and addresses of the patients. Those who claimed “research protection” were not approached. It proved difficult both for the departments and the DNPR to separate identification of IDD‐diagnosed patients in the study period (1998–2008) from patients diagnosed outside of this period, possibly leading to unequal sampling (Asgari et al., 2011). The study was designed as a population‐based historical cohort study with information from multiple sources, and subjects with overlapping information were contacted via a questionnaire (response rate 70%) (Asgari et al., 2011). The questionnaire was designed for MS, TM, and ON diagnosis, respectively, and the patients had the opportunity to provide information on their disease. A total of 477 patients were ascertained for close evaluation, including patients who had not been seen regularly at the departments of neurology or ophthalmology after IDD diagnosis, and nursing home residents (Asgari et al., 2011). The final population was established based on the inclusion criteria of the NMO/NMOSD diagnostic criteria 2006 (Wingerchuk et al., 2006), episodes of ON and/or TM, and an initial brain MRI (obtained within the first year of the onset of symptoms) that did not meet the diagnostic criteria for MS at disease onset (McDonald dissemination in space criteria) (Asgari et al., 2011). The diagnoses were established during the investigated decade (Asgari et al., 2011). A total of 163 patients (86 MS, 5 NMO, 44 ON, 28 TM) fulfilled the inclusion criteria (Asgari et al., 2011). Of these, 35 patients received natalizumab treatment, which according to general treatment guidelines were given to MS patients with high disease activity (Asgari et al., 2011) and are not recommended as treatment for NMOSD (Gahlen et al., 2017; Kleiter, Hellwig, & Berthele, 2012). It could be concluded that the population represented IDD patients with a high risk for NMOSD. The design of the diagnostic algorithm for NMOSD included clinical, radiological, and serological examination performed as independent processes (Asgari et al., 2011). The clinical diagnosis was established without knowledge of AQP4‐IgG results and vice versa, diminishing bias in the study (Asgari et al., 2011). All MRIs during follow‐up were reevaluated blind by a neuroradiologist, and supplementary brain MRI (in 58 patients) and spinal MRI (in 108 patients) scans were taken if missing or if a relapse had occurred since the last MRI (Asgari et al., 2011). Visual evoked potential (VEP) was performed with all patients, facilitating recognition of clinical/subclinical VEP abnormalities (Asgari et al., 2011). In this study, AQP4‐IgG was detected with a recombinant immunofluorescence assay using HEK293 cells transfected with recombinant human full‐length AQP4 gene. MS patients were used as disease controls in addition to healthy controls (Asgari et al., 2011). None of the disease controls nor the healthy controls were positive for AQP4‐IgG (Asgari et al., 2011; Asgari, Nielsen, Stenager, Kyvik, & Lillevang, 2012). Specificity of this assay was validated later in a multicenter study (Waters et al., 2016). A total of 42 patients qualified for the diagnosis of NMO/NMOSD. Twenty‐six (62%) of these were AQP4‐IgG–positive (Asgari et al., 2011). In the seropositive group, antibody positivity was necessary to confirm the diagnosis in 15 cases (36%), whereas 27 (64%) could be diagnosed solely on clinical criteria.

The clinical phenotype was similar to the findings of previous studies (Asgari et al., 2011). The abnormalities on CNS MRI were described in several reports with a focus on the brainstem and spinal cord (Asgari et al., 2017; Asgari, Skejoe, & Lennon, 2013; Asgari, Skejoe, Lillevang, et al., 2013). The diagnostic suggestions and documentation of the clinical, radiological, and serological data on all diagnosed patients with NMO/NMOSD were sent to their respective centres for close follow‐up and treatment. The yearly incidence rate of NMO/SD was estimated to be 0.4 per 10^5^ person‐years (95% confidence interval [CI] 0.30–0.54) and the prevalence was 4.4 per 10^5^ (95% CI 3.1–5.7).

Scrutinizing the data from this population‐based study, two important variables should be considered: the calculation of the incidence rate and the definition of the population. The incidence rate was based on a diagnosis of NMOSD within the study period, not the stipulated disease onset. Thus, the incidence rate was based on NMOSD patients who had a diagnosis of IDD within the study period. Five AQP4‐IgG–positive NMOSD patients and one seronegative NMOSD patient did not have clinical onset of NMOSD within the study period. The five regions in Denmark were established on 1 January 2007, and therefore some IDD patients from the present region of Central Denmark were part of the population in the former Vejle County, now in the region of Southern Denmark. A total of five AQP4‐IgG–positive NMOSD patients in this study originated from the present region of Central Denmark. Based on this information, the cohort in this study consisted of 16 AQP4‐IgG–positive NMOSD patients with onset in the period 1998–2008. The yearly incidence rate was estimated to be 0.15 per 10^5^ person‐years (95% CI 0.13–0.18) and the prevalence 1.68 per 10^5^ person‐years (95% CI 0.86–2.5). A total of 31 NMOSD patients (16 seropositive and 15 negative) were identified with onset in the period 1998–2008, resulting in an incidence of 0.30 per 10^5 ^person‐years (95% CI 0.26–0.33) and a prevalence of 3.26 per 10^5 ^person‐years (95% CI 2.1–4.4). The prevalence and incidence estimates were comparable to that of a population‐based study in Olmsted County, Minnesota, USA (Flanagan et al., 2016), which compared the population‐based seroprevalence and seroincidence of AQP4‐IgG autoimmunity among patients with an IDD in two ethnically divergent populations (2003–2011) (Flanagan et al., 2016). It is generally agreed that the diagnostic certainty is lower for NMOSD patients who are negative for AQP4‐IgG. In some seronegative cases, AQP4‐IgG test results can turn positive when repeated with a second, methodologically independent assay (Wingerchuk et al., 2015) or when retesting is done during an acute attack or at treatment‐free intervals (Juryńczyk et al., 2016). Therefore it is important to have long‐term follow‐up to reevaluate the clinical status and diagnosis (Juryńczyk et al., 2016). These patients may have other autoantibodies, such as MOG‐IgG (Jarius, Kleiter, Ruprecht, et al., 2016). In collaboration with others, the authors subsequently observed that MOG‐IgG is present in a subset of previously reported AQP4‐IgG–negative NMOSD patients (Jarius, Kleiter, Ruprecht, et al., 2016; Jarius et al., 2016a, 2016b; Pache et al., 2016).

In conclusion, the study provided data on the prevalence and incidence of NMO/NMOSD in a predominantly Caucasian population. The strength of the epidemiological and clinical aspects of the study was the diagnostic algorithm for NMO/NMOSD. The review of serological data was done blind, facilitating an analysis of the diagnostic accuracy of AQP4‐IgG. Furthermore, supplementary MRIs were taken if missing or if a relapse had occurred since the last MRI and all MRIs were reevaluated blind by a neuroradiologist. The study indicated that NMO/NMOSD is more common in a Caucasian population than earlier believed. As a consequence, NMO/NMOSD may be considered a more obvious differential diagnosis than previously thought in diagnostic algorithms for MS as well as for other CNS IDDs. In cases with positive AQP4‐IgG, the diagnosis was possible with any of the NMOSD characteristic clinical episodes described above, whereas a negative serological AQP4‐IgG test left some uncertainty.

### 1.4 2007–2014

A decade later, Papp and colleagues (Papp et al., 2018) studied the incidence and prevalence of NMOSD in Denmark with an 8‐year observation period (1 January 2007 to 31 December 2014). The author (Papp et al., 2018) obtained information from tertiary hospitals combined with laboratory databases on the determination of AQP4‐IgG to estimate the incidence and prevalence of NMOSD in Denmark. AQP4‐IgG measurement became available in December 2007 and therefore this data source was not available for the previous study (Asgari et al., 2011). The Danish MS registry was searched for ascertainment of cases (Papp et al., 2018). The number of MS cases with ON or TM was surprisingly low, with approximately 1.9% (300/15848) of patients alive (Papp et al., 2018). The incidence rate for MS in Denmark is estimated to be 12.3 per 100,000 in women and 6.1 per 100,000 in men (Koch‐Henriksen, Thygesen, Stenager, Laursen, & Magyari, 2018). ON is a frequent early inflammatory demyelinating event of MS and NMOSD, and 50%–60% of MS patients and 90% of NMOSD patients will have at least one episode of ON during the course of disease (Matiello et al., 2008; de Seze et al., 2008). Recently, the age‐specific incidence rate of ON in Denmark was estimated at 3.28/100.000‐person‐years (4.57 for women and 2.02 for men) (Soelberg et al., 2017). During an observation period of 8 years (as in the Papp et al. study) roughly 5,000 will receive a diagnosis of MS and 1,000 a diagnosis of ON. An explanation for the low number of ON diagnoses might be that not all ON patients were evaluated because medical care for patients with ON is primarily delivered by ophthalmologists. Furthermore, in the relevant time period (2007–2014) patients with primary or secondary progressive forms of MS may not be followed by a neurology department. Moreover, in contrast to the previous study (Asgari et al., 2011), Papp et al. (2018) excluded patients who were not seen at a neurology department for the last 5 years. Papp et al. (2018) explained that this was unexpected in NMOSD, given the severity of this condition. However, patients who died may have been excluded. The other data source for the MS Registry is the DNPR, which has limitations (Asgari et al., 2011; Papp et al., 2018). In the previous study (Asgari et al., 2011) the Danish MS registry was asked for ascertainment of cases but this was not possible due to updating of the MS registry database at the start of the study. There is agreement in both studies (Asgari et al., 2011; Papp et al., 2018) that the MS Registry and DNPR registry were strongly mutually dependent and also dependent on the departments, acting as a single source of information (Papp et al., 2018). The assay methodology for the detection of AQP4‐IgG is important, as sensitivities vary broadly, particularly with regard to the differential diagnosis of NMOSD versus MS (Waters et al., 2016). Papp et al. utilized laboratory databases which provided AQP4‐IgG determination from different AQP4‐IgG assays, including enzyme‐linked immunosorbent assay, cell‐based assay (CBA) and immunoprecipitation assay, which may have influenced their results (Papp et al., 2018). A small fraction of the samples were retested with CBA at the John Radcliffe Hospital (Oxford, UK) (Papp et al., 2018). However, a direct comparison of the accuracy of the different assays (e.g., based on a ring test principle on the same samples [Waters et al., 2016]) was not done. Patients were selected based on one of the following criteria: (a) at least one positive AQP4‐IgG test result; (b) NMOSD diagnosis; or (c) suspicion of NMOSD based on the 2015 IPND criteria. The authors excluded 98.3% of cases after review of records due to lack of documentation of the NMOSD core clinical characteristics (Dale et al., 2018), without confirmation via questionnaire, interview, clinical exam or MRI, VEPs, or retesting AQP4‐IgG during an acute attack (Papp et al., 2018). Only patients with the two most common clinical characteristics of seropositive NMOSD (a history of ON or TM) were identified (Papp et al., 2018). The other four core symptoms are: area postrema syndrome; symptomatic narcolepsy; acute diencephalic clinical syndrome with NMOSD‐typical diencephalic MRI lesions; and symptomatic cerebral syndrome with NMOSD‐typical brain lesions (Papp et al., 2018). Brainstem symptoms and signs occur in almost one‐third of NMOSD patients and even more frequently in AQP4‐IgG–positive patients (Kremer et al., 2014), suggesting brainstem involvement as an important diagnostic marker of NMOSD (Asgari, Skejoe, & Lennon, 2013; Zekeridou & Lennon, 2015). These patients will need closer investigation through clinical evaluation and supplementary MRIs. For 16 cases (that were AQP4‐IgG–positive by CBA), the status was later determined via new serum samples. Fourteen cases became seronegative and these cases were excluded (Papp et al., 2018). As the patient's subsequent serostatus may be influenced by immunosuppressive treatment, disease stage and severity, and variation in assay techniques, such a maneuver should preferably be performed on the same samples (Melamed et al., 2015). Papp et al. (2018) raised a number of critical red flags for false‐positive NMOSD diagnosis in low AQP4‐IgG–positive patients by CBA, such as spinal cord lesion shorter than three segments, the presence of oligoclonal bands (OCBs), and more brain MRI‐specific findings, which led to exclusion from the study (Papp et al., 2018). The dynamic formation of LETM (cord lesion extending three or more vertebral segments) on spinal cord MRIs (Asgari, Skejoe, & Lennon, 2013) and modifications of LETM into multiple shorter lesions and atrophy have been observed in the course of NMOSD (Asgari, Skejoe, Lillevang, et al., 2013), in particular due to treatment and remission. This indicates that the timing of the MRI of the spinal cord may be important for the demonstration of LETM (Wingerchuk et al., 2006). Additionally, short TM has been shown (Flanagan et al., 2015) not to be uncommon in NMOSD and, when present, may delay diagnosis and treatment. Short MRI lesions occurred at least once in the disease course in 15%–36% of AQP4‐IgG–positive NMOSD patients (Jarius et al., 2018). With regard to the presence of OCBs, 15%–30% of NMOSD patients have OCBs (Jarius et al., 2011), which are especially transitory during attacks (Weinshenker & Wingerchuk, 2017). Other cerebrospinal fluid (CSF) findings in AQP4‐NMOSD may mimic infectious TM with neutrophil pleocytosis and impaired blood‐CSF barrier function (Weinshenker & Wingerchuk, 2017). Collectively, the complexities associated with use of AQP4‐IgG as a biomarker in NMOSD emphasize the importance of optimization and standardization of AQP4‐IgG assays and clinical diagnostic criteria.

Papp et al. (2018) identified 56 patients with a diagnosis of NMOSD according to the 2015 criteria (Wingerchuk et al., 2015). Of these, only 27 patients had onset of disease in the period 2007–2013, resulting in an incidence of NMOSD of 0.070 per 100,000 person‐years (95% CI 0.0463–0.1022) (Papp et al., 2018) with a prevalence rate of 1.090 (95% CI 0.808–1.440). Out of the 56 NMOSD cases that the authors identified, 54 cases (96%) originated from the laboratory databases and 46 cases originated (82%) from neurology departments (Papp et al., 2018). In summary, a limitation in this study was that data were collected from a subset of the study population based on AQP4‐IgG positivity from laboratory testing and included only current or recently active NMOSD cases, who had been seen in MS clinics or undergone AQP4‐IgG testing. The strength of the study was the use of laboratory databases of AQP4‐IgG determinations as a new source of information.

## 2 CONCLUSION

Differences in the definition and accuracy of NMOSD diagnosis, the completeness of case ascertainment, and variability in assays for AQP4‐IgG should be considered when evaluating epidemiological studies of NMOSD. Data from the two epidemiological studies (1998–2008 and 2007–2014) reflect the complexity of NMOSD. However, there has in the last decades been an increased recognition of NMOSD as a distinct entity, which may facilitate recommendation consensus and provide data for further studies based on the Strengthening the Reporting of Observational Studies in Epidemiology (STROBE) statement. We need to address whether some AQP‐IgG–positive patients become seronegative during the course of their disease and vice versa. Currently, the classification of AQP4‐IgG–negative NMOSD patients may require new diagnostic categories. Further multicenter studies applying standardized definitions and methodologies are needed to search for new antibody targets in clinically well‐defined NMOSD patients who are both AQP4‐IgG and MOG‐IgG–negative. Very recently, a novel CNS disorder with glial fibrillary acidic protein (GFAP) IgG as a biomarker was described. The disorder involves the meninges, optic nerve, brain, and spinal cord, and has been termed meningo‐encephalomyelitis (Flanagan et al., 2017). Notably, spinal cord imaging frequently demonstrated LETM with area postrema involvement in autoimmune GFAP astrocytopathy (Flanagan et al., 2017; Sechi & Flanagan, 2019). This may mimic AQP4‐IgG and MOG‐IgG–related autoimmunity or seronegative NMOSD. The classical 1894 definition of NMO originates from Gault and Eugene Devic (Devic's disease). Based on 17 cases, it characterized NMO as an acute, fulminant, monophasic disorder consisting of ON and TM occurring simultaneously or in rapid succession (E. D., 1894). This classical NMO definition may today segregate into AQP4‐IgG, MOG‐IgG and GFAP‐related autoimmunity or double seronegative NMOSD patients. We need to support this development in collaboration networks, preferably via multicenter investigations. Specifically, we need adequately powered prospective multicenter epidemiological studies that apply standardized methodologies to follow the natural course of these diseases in order to draw more specific conclusions.

## CONFLICT OF INTEREST

None.

## Data Availability

For the study reported in Asgari et al. (2011), clinical data and biological material were registered within the Odense Patient data Explorative Network (the OPEN database) to enable future collaborate projects.
